# SHTXTHHly, an extracellular secretion platform for the preparation of bioactive peptides and proteins in *Escherichia coli*

**DOI:** 10.1186/s12934-022-01856-8

**Published:** 2022-06-27

**Authors:** Wen Zhu, Yang Wang, Liangyin Lv, Hui Wang, Wenqiang Shi, Zexin Liu, Wei Yang, Jianwei Zhu, Huili Lu

**Affiliations:** 1 Engineering Research Center of Cell and Therapeutic Antibody, Ministry of Education, School of Pharmacy, Shanghai Jiao Tong University, 800 Dongchuan Road, Shanghai, 200240 China; 2 Institute of Chinese Materia Medica, China Academy of Chinese Medical Sciences, Beijing, 100700 China

**Keywords:** Hemolysin A, *E. coli*, Secretory expression, S tag, Rapid bioactivity detection

## Abstract

**Background:**

In previous work, we developed an *E. coli* extracellular secretion platform XTHHly based on the hemolysin A secretion system. It can produce bioactive peptides with simple purification procedures. However, the wider application of this platform is limited by poor secretion efficiency.

**Results:**

In this study, we first discovered a positive correlation between the isoelectric point (pI) value of the target protein and the secretion level of the XTHHly system. Given the extremely high secretion level of S tag, we fused it at the N-terminus and created a novel SHTXTHHly system. The SHTXTHHly system significantly increased the secretion levels of antimicrobial peptides (PEW300, LL37, and Aurein 1.2) with full bioactivities, suggesting its excellent capacity for secretory production of bioactive peptides. Furthermore, RGDS, IL-15, and alcohol dehydrogenase were successfully secreted, and their bioactivities were largely maintained in the fusion proteins, indicating the potential applications of the novel system for the rapid determination of protein bioactivities. Finally, using the SHTXTHHly system, we produced the monomeric Fc, which showed a high affinity for Fcγ Receptor I and mediated the antibody-dependent immunological effects of immune cells, demonstrating its potential applications in immunotherapies.

**Conclusions:**

The SHTXTHHly system described here facilitates the secretory production of various types of proteins in *E. coli*. In comparison to previously reported expression systems, our work enlightens an efficient and cost-effective way to evaluate the bioactivities of target proteins or produce them.

**Graphical abstract:**

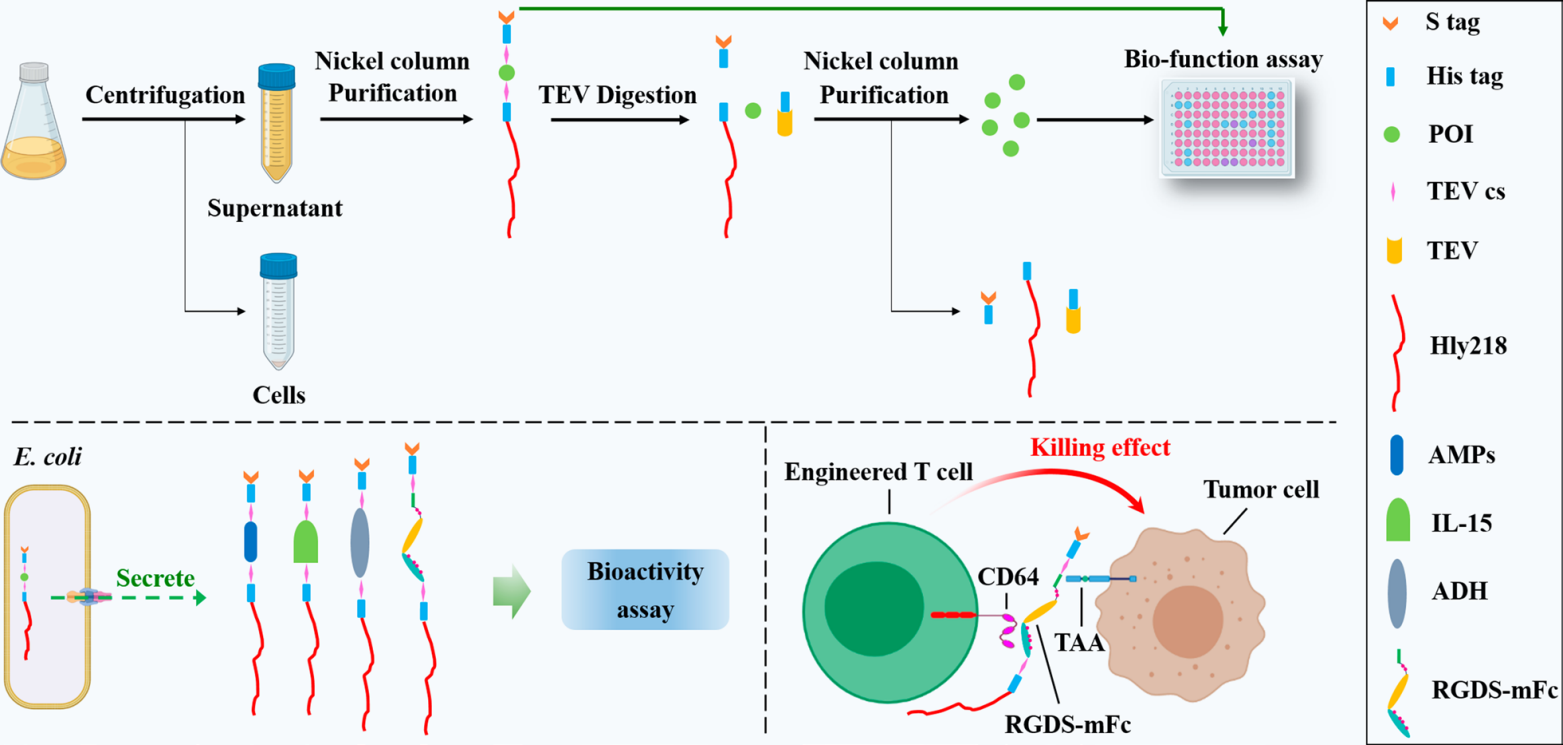

**Supplementary Information:**

The online version contains supplementary material available at 10.1186/s12934-022-01856-8.

## Background

*Escherichia coli* (*E. coli*) retains its popularity as the most versatile system for producing recombinant proteins [1]. As opposed to cytoplasmic expression, extracellular secretion of recombinant proteins in *E. coli* can avoid inclusion body formation, provide a better folding environment free from proteolytic degradation, and facilitate simple detection and easy purification [2]. Among the many secretion systems in *E. coli*, the hemolysin A (HlyA) secretion system has received much attention for its one-step secretion property [3]. Protein of interest (POI) fused to the C-terminal signal sequence of HlyA can be recognized and secreted directly with the aid of two accessory proteins, hemolysin B (HlyB) and hemolysin D (HlyD) [4]. In our previous study, we designed the XTHHly production system, which comprised POI, tobacco etch virus enzyme cleavage site (T), 6*His tag (H), and the C-terminal 218 amino acids of HlyA, and verified its use in the production of tag peptides and antimicrobial peptides (AMPs) [5]. Though these peptides can be obtained rapidly and cost-effectively with bioactivities comparable to chemically synthetic ones, the yields of some peptides were not satisfying (less than 1 mg/L). The system must be further improved before envisaging practical applications for academic and industrial purposes.

Tag fusion is an effective approach for recombinant protein production owing to its excellent performance to increase expression, improve solubility, help refolding, and simplify purification [6]. Varieties of peptides, proteins, and their derivatives have been developed and used as fusion tags to date [7]. Some tags, such as histidine tag, can help achieve a higher level expression for a poorly expressed protein, as well as assist the efficient purification of target proteins [8]. N-utilization substance A (NusA) [9], Thioredoxin (Trx) [10], Disulfide isomerase I (PDI) [11], Cellulose binding domain (CBD) [12], and superfolder green fluorescent protein (sfGFP) [13] are among the other available expression enhancer tags. It is anticipated that the XTHHly system could be further optimized by fusion to proper tags.

*E. coli* is a suitable tool for rapid protein production and screening due to its easy genetic manipulation, inexpensive culture, and fast growth. Rapid procedures to screen for expression and solubility of recombinant proteins or to produce and characterize antimicrobial colicins were described using an *E. coli* cell-free extract [14, 15]. A novel di-cistronic auto inducible system was used to rapidly screen high expressing *E. coli* colonies, and screening times were shortened from several days to 18 h [16]. In this respect, secretory expression provides considerable advantages. There is no need to prepare cell-free extract or couple the target proteins with reporter proteins since bioactive proteins can be detected directly in the supernatants. Up to now, researches about *E. coli*’s secretion pathways mainly focus on toxins assay, and only scattered studies have applied them to rapid bioactivity determination.

In this work, we firstly demonstrated that the secretion levels positively correlated to the isoelectric point (pI) values of the target proteins and reported that the N-terminus fused S tag significantly increased the efficiency of the HlyA secretion system, allowing us to develop a novel SHTXTHHly system. This system produced three AMPs at considerable levels and was verified to possess similar activities to the chemically synthetic ones. The bioactivities of RGDS, IL-15, and alcohol dehydrogenase (ADH) can be detected in the SHTXTHHly fusion form with the supernatants directly, which encouraged us to explore more applications of this system. Finally, we demonstrated that monomeric Fc, whether in the fusion form or not, can bind to Fcγ receptor I (CD64) and mediate CD64 chimeric effector cells’ immunological outputs, suggesting the great potential of bacteria-produced antibodies. Overall, these results highlight the broad range of applications of the SHTXTHHly system.

## Results

### The positive correlation between the pI value and the secretion level

**Fig. 1:**
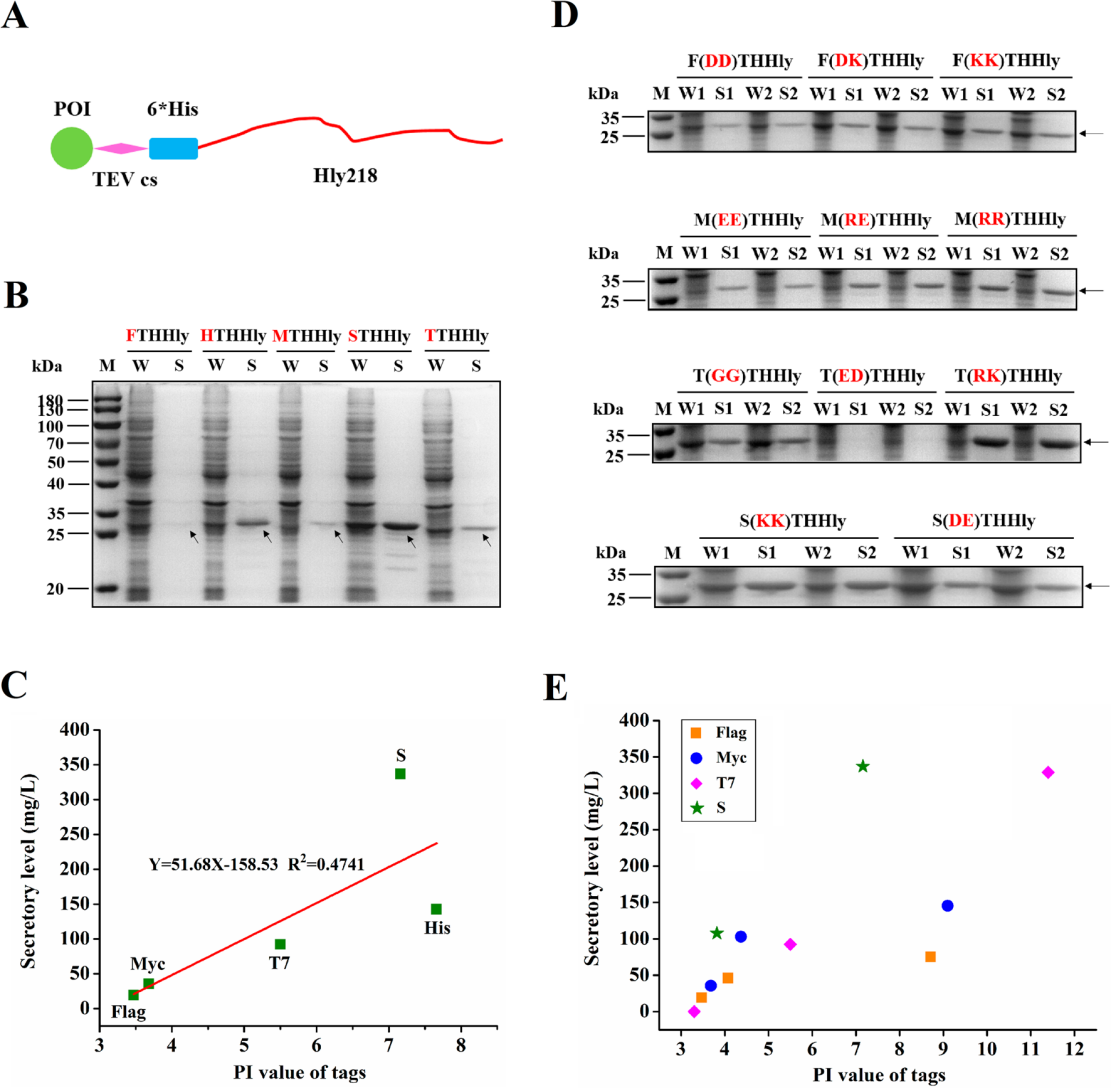
Positive correlation between the pI value and the secretion level. **A** Schematic diagram of the XTHHly system. POI: Protein of interest; TEV cs: TEV enzyme cleavage site; Hly218: the last 218 amino acids of hemolysin A. **B**–**D** SDS-PAGE analysis of culture samples (n = 2). W: Whole-cell lysate; S: Supernatant. The native and mutated tag fusion proteins are indicated above the image. The arrows indicate the tag fusion proteins. **C–E** The relationship between the pI value and the secretion level. The secretion levels of mutated tag fusion proteins were estimated by Image J scanning. All SDS-PAGE was conducted under non-reducing conditions

We previously developed the XTHHly platform, which could be used for the secretory production and simple purification of peptides (Fig. 1A) [5]. To further improve the efficiency of this system, here we screened the secretion levels of the model peptides, including Flag tag, His tag, Myc tag, S tag, and T7 tag, by induction for 4 h with arabinose and another 4 h with isopropyl-β-D-thiogalactoside (IPTG) (Fig. 1B). The tag fusion proteins were also expressed intracellularly by omitting the arabinose addition step. Only the expression of THHly and STHHly can be detected, and the levels were much lower than the secretory expression groups (Additional file 1: Fig. S1). Interestingly, a linear correlation (R^2^ = 0.47) between the pI value and the secretion level was observed (Fig. 1C). To investigate the effect of protein charge on the secretion level, a series of amino acid substitutions that changed the pI values were introduced. At constant pH, proteins with higher pI values carry more positive charges. In all four cases, positive charges greatly promoted the secretion of the proteins (Fig. 1D). They increased the total expression levels (for Flag tag and Myc tag) or the secretory efficiency (for T7 tag). On the contrary, if acidic amino acids were introduced, the secretion levels decreased dramatically (for S tag) or even were completely abolished (for T7 tag). In summary, the side-by-side comparisons reveal a positive relationship between the pI value and the secretion level (Fig. 1E).

### Promotion effect of S tag on protein secretion

**Fig. 2:**
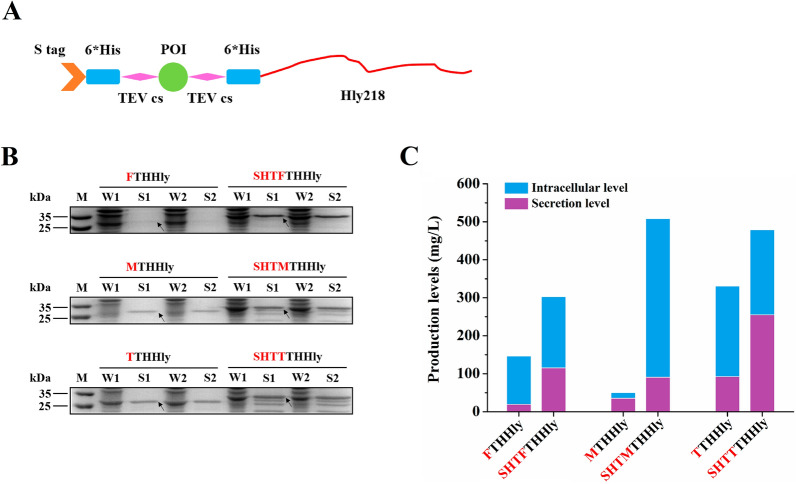
Promotion effect on the secretion of S tag. **A** Schematic diagram of the SHTXTHHly system. POI: Protein of interest; TEV cs: TEV enzyme cleavage site; Hly218: the last 218 amino acids of hemolysin (**A)**. **B** SDS-PAGE analysis of culture samples (n = 2). W: Whole-cell lysate; S: Supernatant. The native and S tag-fused tag fusion proteins are indicated above the image. The arrows indicate the tag fusion proteins. **C** Summary of the expression profiles of the tag fusion proteins. The expression levels were estimated by Image J scanning. All SDS-PAGE was conducted under non-reducing conditions

Given the medium pI value but relatively high secretion level, S tag stands out among the five tags. To investigate whether S tag can promote the expression or secretion capacities of the XTHHly system, we designed an advanced construct by fusing S tag, 6*His tag, and TEV cleavage site to the N-terminus, that is, the SHTXTHHly system (Fig. 2A). Such a design is reasonable because the nickel column can catch the un-cut fusion proteins, the digested fragments, and the His tag conjugated TEV enzyme, and only the target proteins would be flowed through. After N-terminus fusion with SHT, all the studied tags showed obviously higher secretion levels (Fig. 2B). Quantitative analysis of the sodium-dodecyl sulfate–polyacrylamide gel electrophoresis (SDS-PAGE) gels was performed with Image J, and the results were summarized in Fig. 2C. These results suggest that the fused S tag significantly increased the secretion levels by promoting either protein expression (for Flag, Myc, and T7 tags) or protein secretion (for Flag and T7 tag).

### SHTXTHHly system improves secretory production of bioactive AMPs

**Fig. 3:**
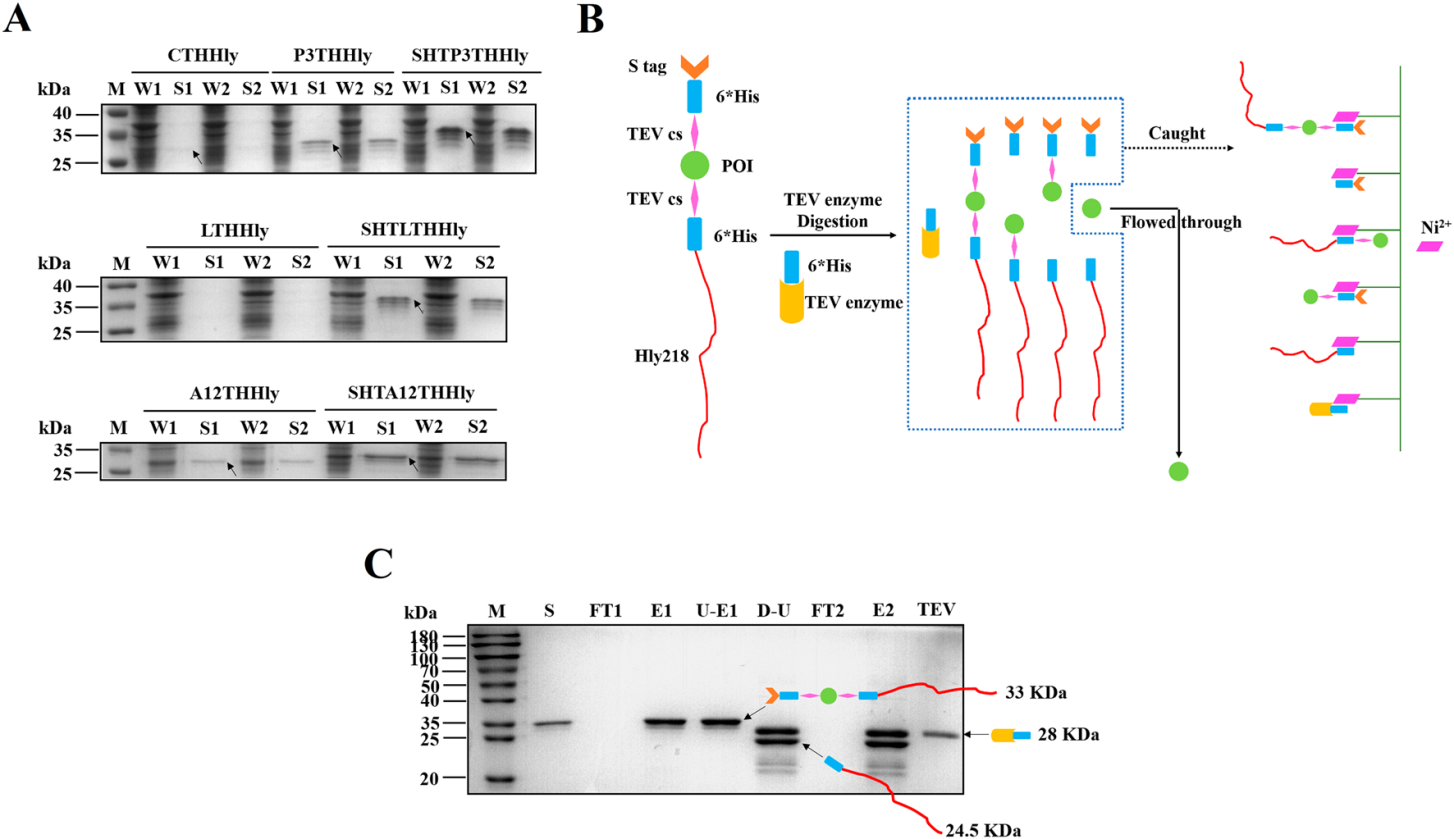
Production of the antimicrobial peptides using the SHTXTHHly system. **A** SDS-PAGE analysis of culture samples (n = 2). W: Whole-cell lysate; S: Supernatant. The native and S tag-fused tag fusion proteins are indicated above the image. The arrows indicate the tag fusion proteins. **B** Schematic diagram of the purification procedure. After TEV enzyme digestion, the un-cut fusion proteins, the digested fragments, and the His tag conjugated TEV enzyme would be caught by the nickel column, and only the target proteins would be flowed through. **C** SDS-PAGE analysis of the purification of PEW300. S: Supernatant; FT: Flow-through; E: Eluent; U-E: Ultra-filtration product of the eluent; D-U: TEV enzyme digestion production of the ultra-filtration product; 1: First round nickel affinity chromatography; 2: Second round nickel affinity chromatography. All SDS-PAGE was conducted under non-reducing conditions

Based on the results above, we next explored the application of the SHTXTHHly system for the production of bioactive peptides. PEW300 (P3), a novel AMP designed with three mutations to the parental peptide Cecropin A (CeA), possessed a higher pI value and more positive charges (pI value: P3 11.51; CeA 10.39). Compared with CTHHly, the clear band corresponding to P3THHly in the supernatant confirmed the promotion effect of positive charges (Fig. 3A). The N-terminus fused S tag further increased the secretion to a considerable level, and such an effect was also observed for LL37 and Aurein 1.2. The levels of the fusion proteins in the supernatant were 75.41 (SHTP3THHly), 40.82 (SHTL3THHly), and 139.59 (SHTA12THHly) mg/L, respectively.

The technical map of TEV enzyme digestion and purification is shown in Fig. 3B. The fusion proteins in the supernatants were purified by one-step nickel affinity chromatography and concentrated by ultrafiltration (Fig. 3C, only the purification of SHTP3THHly is shown). After His tag-conjugated TEV enzyme digestion, the AMPs were released from the fusion proteins, obtained in the flow-through (FT) of the secondary nickel affinity chromatography, and concentrated with an ultrafiltration tube. The bands with slightly lower molecular weights than the target proteins had been proved to be the C-terminus degraded fragments in our previous work. They would not cause trouble since the fragments released from the degraded fusion protein can also be caught by the secondary nickel affinity chromatography. In summary, 4.42 mg PEW300, 2.84 mg LL37, or 4.14 mg Aurein 1.2 can be obtained from one-liter medium, which was much higher than those obtained by the XTHHly system. These results show that the SHTXTHHly system can produce peptides in a more time- and cost-effective way.

### Determination of the antimicrobial/antitumor activity of rP3/rL3/rA1.2

**Fig. 4:**
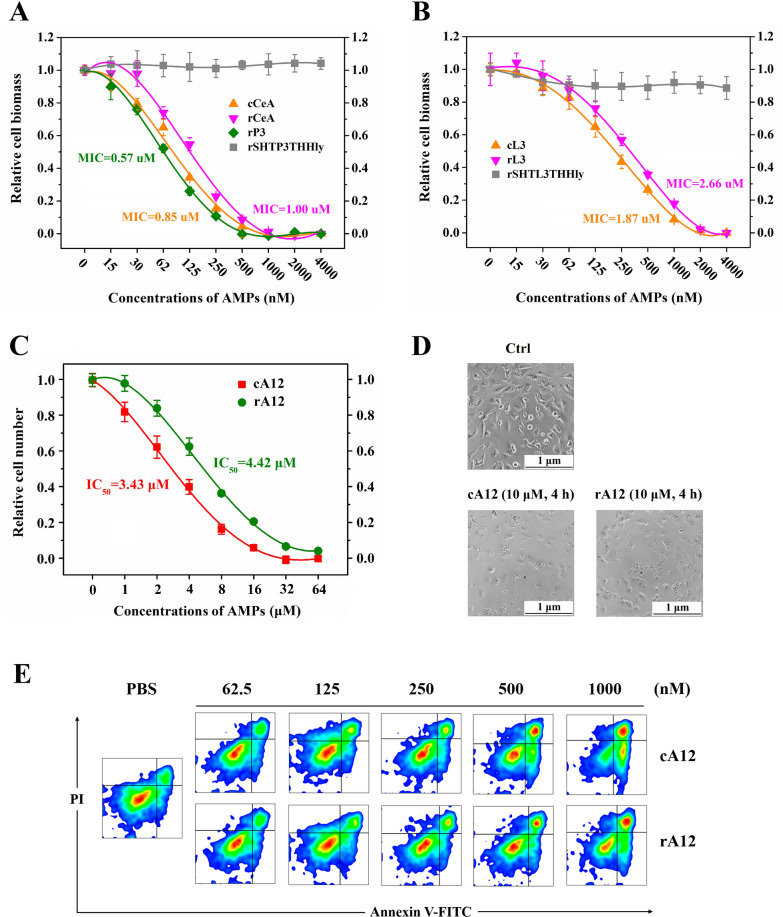
Bioactivities determination of the produced AMPs. Different concentrations (0–4000 nM) of cCeA and rCeA (**A**), or cLL37 and rLL37 (**B**), were incubated with *E. coli* 25,922 at 37 °C for 18 h, then the OD_600_ values were detected. Different concentrations (0–64 μM) of cA12 and rA12 (**C**) were incubated with U87 MG cells for 8 h, and the number of live cells was measured with the CCK-8 reagent. In other experiments, the concentration of A12 and the incubation time were set at 10 μM and 4 h, then the tumor lysis (**D**) and pro-apoptosis (**E**) effect were verified with a fluorescence microscope or an Annexin V-FITC/PI dye

After TEV enzyme cleavage, the peptides released from the fusion protein contained extra N-terminal glycine and six C-terminal amino acid residues. To investigate the impact of these extra residues on the bioactivities, we compared the recombinant peptides produced by the SHTXTHHly system with chemically synthesized ones side-by-side. The minimum inhibitory concentration (MIC) of rCeA turned out to be 1.00 μM, higher than that of cCeA (0.85 μM) and rP3 (0.57 μM) (Fig. 4A). Such a variation was also seen between rL3 (MIC: 2.66 μM) and cL3 (MIC: 1.87 μM) (Fig. 4B). No antimicrobial activity was detected for the fusion proteins due to the blocking effect of the N-terminal SHT and C-terminal HlyA, implying that the AMPs would not affect the cells during the production process. Besides, rP3 and rL3 retained good antimicrobial activities after autoclaving, displaying their attractive thermal stability compared with traditional antibiotics (Additional file 1: Fig. S2). For the antitumor activity of Aurein 1.2, the IC_50_ of rA12 to U87 MG cells was 4.42 μM, higher than that of cA12, 3.43 μM (Fig. 4C). At 10 μM, rA12 exhibited similar tumor lysis (Fig. 4D) and pro-apoptosis effect (Fig. 4E) to cA12. These results prove that the AMPs produced by the SHTXTHHly system possess comparable bioactivities with chemically synthetic commercial products.

### Applications of the SHTXTHHly system in the rapid determination of target protein bioactivity

**Fig. 5:**
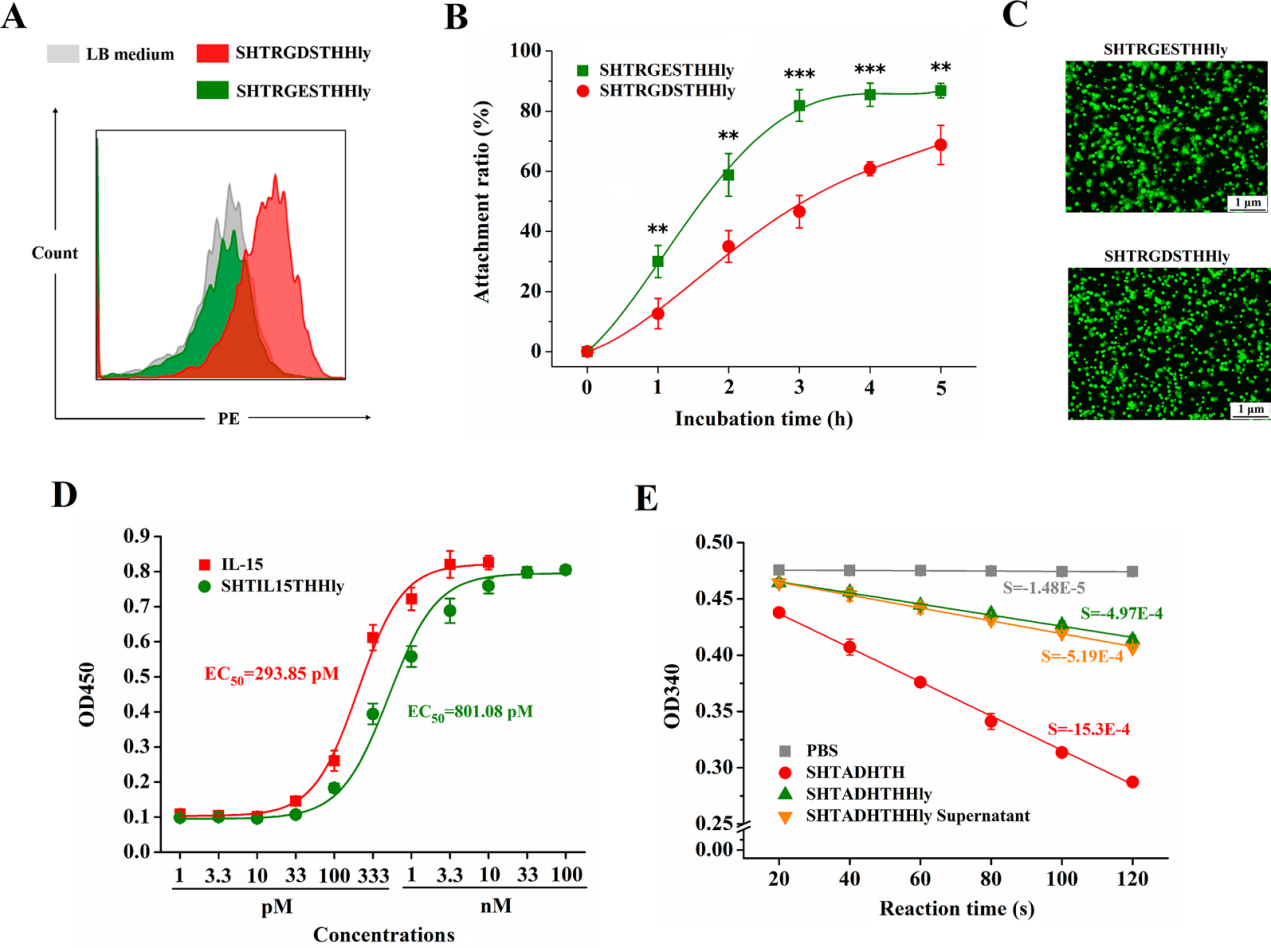
Bioactivities determination of RGDS, IL-15, and ADH in the SHTXTHHly fusion form. U87 MG cells were incubated with SHTRGDSTHHly/SHTRGESTHHly, mouse anti-S tag antibody, and the second antibody in sequence, then the cell staining was measured by FACS (**A**). U87 MG cells were incubated with SHTRGDSTHHly/SHTRGESTHHly and transferred into a fibronectin-coated plate. At different times, the attached cells were subjected to the CCK-8 reagent for quantification (**B**). In other experiments, cells were stained with DiO dye (green) and observed with a fluorescence microscope (**C**). Mo7e cells were incubated with different concentrations (1 pM-100 nM) of IL-15 and SHTIL15THHly for 4 days, and the cell numbers were measured with a CCK-8 reagent (**D**). The activities of ADH and SHTADHTHHly were compared using Ethyl Acetoacetate as the substrate and NADH as the co-factor (**E**). The activity was measured at 340 nm to monitor the decrease in the absorbance of NADH quantitatively. S: Slope. The 1-tailed Student's t-test was used to determine statistical differences between the two groups

RGDS, IL-15, and ADH were chosen as the model proteins to evaluate the feasibility that the bioactivity of the target protein can be directly detected in the SHTXTHHly fusion form. In the case of the RGDS peptide, both SHTRGDSTHHly and SHTRGESTHHly were secreted at high levels into the supernatants (Additional file 1: Fig. S3A). The RGDS peptide is an integrin recognition motif, and the binding to integrin-expressing U87 MG cells was detected for SHTRGDSTHHly, but not for SHTRGESTHHly (Fig. 5A). Treatment with the supernatant containing SHTRGDSTHHly also obviously inhibited cell attachment (Fig. 5B, C). Therefore, it is feasible to use the SHTXTHHly system to rapidly compare or screen bioactivities of various peptides such as RGDS and its variants.

Under the former induction conditions (37 °C and 220 rpm), no SHTIL15THHly or SHTADHTHHly fusion proteins can be detected in the supernatant by SDS-PAGE, and further analysis revealed that nearly all the proteins formed inclusion bodies (data not shown). Detectable levels of SHTIL15THHly and SHTADHTHHly (13.78 and 26.51 mg/L, respectively) can be achieved after the induction conditions were adjusted to 20 °C and 150 rpm. The proteins were purified, and buffer changed to phosphate-buffered saline (PBS) (Additional file 1: Fig. S3B, S3C), then their bioactivities were investigated. The comparable EC_50_ values of SHTIL15THHly and the control IL-15 (801.08 pM and 293.85 pM) showed that the strong promotion effect on Mo7e cells’ proliferation was still maintained for IL-15 in the fusion form (Fig. 5D). However, SHTADHTHHly demonstrated a much weaker catalytic activity (Fig. 5E). Though weak, the catalytic activity can be tested directly with the crude culture supernatant. Overall, these results indicate that the bioactivities of the target proteins probably could be at least partially preserved in the SHTXTHHly fusion form and thus detected directly with the supernatant.

### SHTXTHHly system facilitates the secretory production of monomeric Fc with the ability to elicit immune responses

**Fig. 6:**
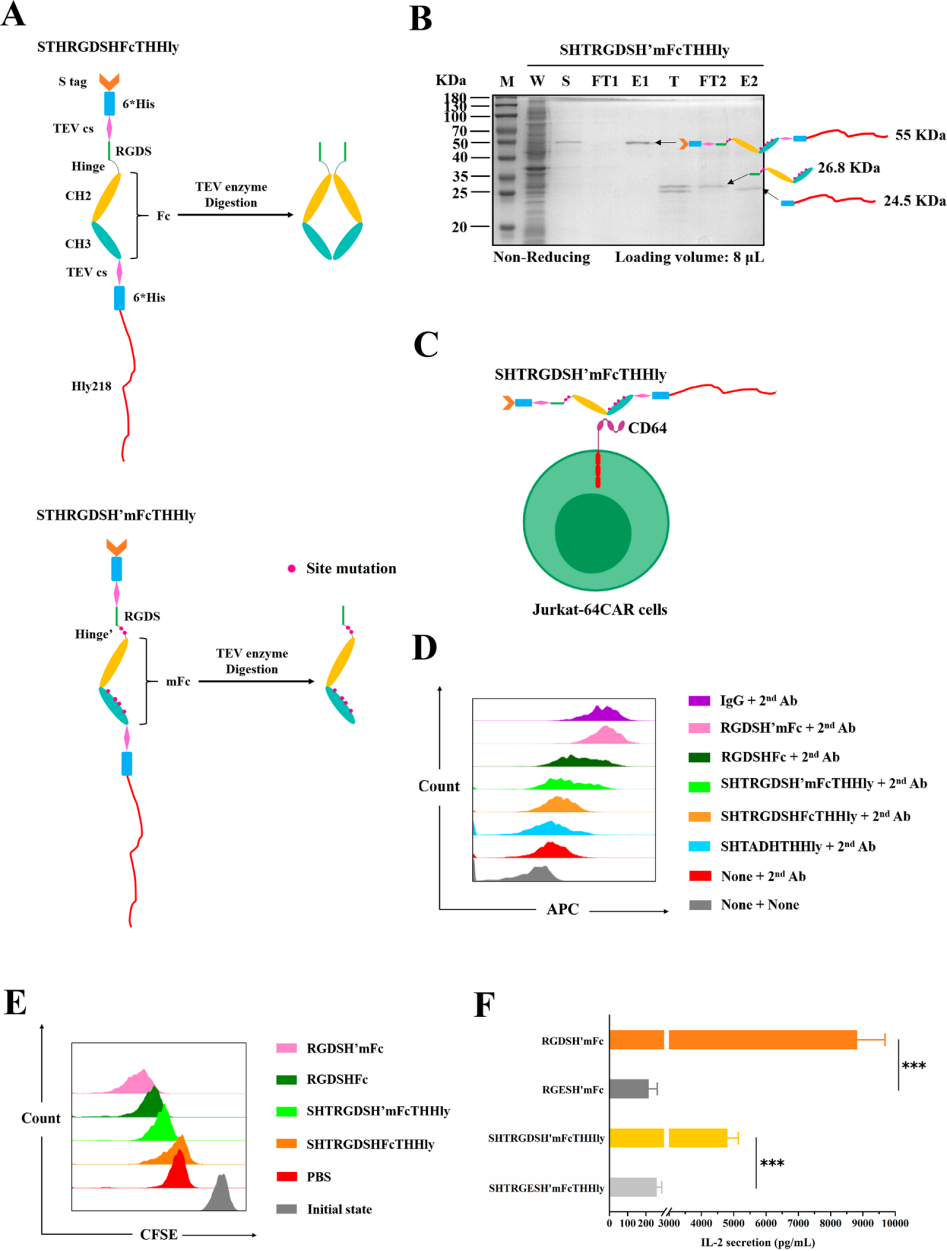
Detection of the cellular outputs mediated by the SHTXTHHly-produced mFc-based antibodies. **A** Schematic diagram of the SHTXTHHly-produced mFc-based antibodies. TEV cs: TEV enzyme cleavage site; H’: Mutated hinge; mFc: Monomeric Fc; Hly218: the last 218 amino acids of hemolysin A. The site mutations were indicted by red points. **B** SDS-PAGE analysis of the purification of RGDSH’mFc. W: Whole-cell lysate; S: Supernatant; FT: Flow-through; E: Eluent; T: TEV enzyme digestion production of the eluent; 1: First round nickel affinity chromatography; 2: Second round nickel affinity chromatography. **C** Schematic diagram of the binding between CD64 chimeric Jurkat cells and the SHTRGDSH’mFcTHHly fusion protein. The red squares indicate the downstream activation elements, namely CD28, CD137 (41BB), and CD247 (CD3ζ). To measure the binding affinities, JK-64CAR cells (2E5) were incubated with target proteins (7 nM) and the second antibody in sequence, and then cell staining was measured by FACS (**D**). U87 MG cells, CFSE-stained JK-64CAR cells, and mFc-based antibodies were incubated for 24 h, then cell activation (**E**) and IL-2 release (**F**) were measured by FACS and the ELISA kit, respectively. The 1-tailed Student's t-test was used to determine statistical differences between the two groups

To explore the potential applications of the SHTXTHHly system in immunotherapy, we constructed SHT-RGDS-Hinge-Fc-THHly and performed six site-mutations (C226S, C229S, L351S, T366R, L368H, and P395K) to obtain a monomeric Fc with an extremely high affinity to Fcγ receptor I (CD64) (Fig. 6A) [17]. The fusion proteins were secreted at about 15 mg/L, and the fragments can be separated after TEV enzyme digestion (Fig. 6B). Unlike RGDSHFc, non-heating RGDSH’mFc migrated as a 26.8 kilo-Dalton (kDa) band on SDS-PAGE, suggesting a monomeric form (Additional file 1: Fig. S4). Jurkat cells chimeric with CD64 outer-membrane domain and downstream costimulatory domain (CD28-CD137-CD247) (JK-64CAR cells) constructed in our lab were used as the effector cells. The binding capacities for CD64 were detected by incubating the proteins with the effector cells (Fig. 6C) and subsequently excessive goat anti-human immunoglobulin G (IgG) antibody (APC). The final APC fluorescence intensity characterized the number of bound proteins, reflecting the affinity to the 64CAR. As shown in Fig. 6D, both SHTRGDSHFcTHHly and SHTRGDSH’mFcTHHly can bind to 64CAR, and the binding was strengthened after TEV enzyme digestion. RGDSH’mFc also bound to 64CAR with high affinity, comparable to native IgG in human serum.

We then investigated whether the binding can mediate JK-64CAR cells’ responses. As described in Methods, we used the produced RGDS-Fc fusions as the adaptors and U87 MG cells as the target to stimulate JK-64CAR cells. In the 4 h cytotoxicity assay, the JK-64CAR cells displayed an affinity-dependent target cell killing effect, and the ADCC effects were low in the absence of the antibodies or JK-64CAR cells (Additional file 1: Fig. S5). The Carboxyfluorescein succinimidyl ester (CFSE) dilution assay also demonstrates a more robust proliferative response mediated by the proteins with higher affinity (Fig. 6E). Finally, JK-64CAR cells produced a high amount of IL-2 in response to the RGDSH’mFc-U87 MG cells or SHTRGDSH’mFcTHHly-U87 MG cells immune complexes, but not the RGES controls (Fig. 6F). The results convinced that the SHTXTHHly system is an efficient platform to produce monomeric Fc fusions to target antigens and mediate immune responses of CD64-engineered immune cells.

## Discussions

The current study first reported a quantitative correlation between the pI value of the target protein and the secretion level of the HlyA secretion system. The highly-basic and positively-charged peptides were secreted efficiently by the HlyA transporter, whereas peptides with a more negative charge and lower pI value were hardly secreted (Fig. 1). This finding contradicts the finding of Hyunjong et al. who reported that a lower pI value increased signal sequence–mediated secretion of recombinant proteins through a bacterial ATP-binding cassette (ABC) transporter [18]. However, the research merely focused on the LARD3 signal sequence of thermo-stable lipase TliA of *Pseudomonas fluorescens*. Besides pI values, the chosen proteins possessed different sizes (from 30 to 90 kDa), which introduced other enigmatic factors and challenged the conclusion. Comparatively, the site mutations introduced in our work avoid such interference, which facilitated our finding of the promotion effect of positive charge on the secretion of HlyA transporter.

For heterogeneous protein production, tag fusion can improve the expression level or solubility, as well as simplify the downstream purification. Among the several model peptides, S tag, a 15-amino acid peptide derived from pancreatic ribonuclease A, exhibited an extremely high secretion level (330 mg/L). To achieve a higher yield, we fused S tag to the N-terminus of XTHHly to establish a new system named SHTXTHHly. The SHTXTHHly system significantly increased peptides secretion levels, and it was observed that S tag could generally promote secretion efficiency directly or increase total expression level (Fig. 2). This finding broadly supports other studies in which S Tag fusion can help overexpress NtrX protein [19] or refold human erythropoietin [20]. The mechanisms by which fused tags promote protein expression have been summarized in detail elsewhere, including stabilizing mRNA structure, aiding protein folding, and enhancing solubility [21]. Solubility is also critical for the secretion system because once the target proteins form inclusion bodies, they can no longer be recognized and transported. Besides, the same N-terminal sequence in SHTXTHHly offers another advantage that the translation initiation complex probably conducts a more stable and efficient translation initiation on the site of S tag [22].

We further explored the SHTXTHHly system’s application in producing bioactive peptides. As small peptides with rapid and broad-spectrum antimicrobial properties, AMPs are promising candidates to complement currently available antibiotics [23]. Since AMPs can disrupt microbial membranes and are liable for proteolytic degradation, they are often produced with a fusion partner in heterologous hosts to neutralize their toxicity and increase their expression levels [24, 25]. Although we previously achieved secretory expression of AMPs, the yields still need to be improved [5]. To address this issue, we used the SHTXTHHly system to express three AMPs and obtained considerable high levels of secretion. In addition, incorporating positively-charged amino acids into the parental CeA peptide not only increased secretion due to the higher pI value but also increased the antimicrobial activity (Fig. 4). All these results suggest that the SHTXTHHly system provides an efficient and cost-effective way to produce large quantities of bioactive peptides or small proteins.

It was reported that the HlyA secretion system could only transport unfolded proteins [26, 27]. In other words, it would be much more difficult to secret large proteins for their complicated tertiary structures. In our study, significantly lower secretion levels of SHTIL15THHly and SHTADHTHHly (13.78 and 26.51 mg/L, respectively) than peptides fusion proteins (~ 100 mg/L for AMPs) confirmed this conception. Nevertheless, from another point of view, it is amenable to assume that the HlyA signal peptide itself possessed a flexible structure. Given that secretory expression immensely facilitates the detection of the target proteins, we were urged to test the bioactivities of the fusion proteins. We started with the RGDS sequence, which can bind to integrin receptors on the cell membrane [28]. The results showed that the HlyA signal peptide would not influence the specific binding (Fig. 5A, B). The promotion effect of SHTIL15THHly on cell proliferation was comparable with monomeric IL-15 (Fig. 5D). However, the catalytic activity of ADH was obviously affected in the fusion form (Fig. 5E). A possible explanation for this might be that, unlike RGDS and IL-15, the bioactivity of ADH is heavily dependent on not only the tertiary structure but also monomer polymerization [29]. Though weaker, the catalytic activity of SHTADHTHHly can be directly detected with the supernatant. Compared with intracellular soluble expression or inclusion body formation where the bacteria lysis procedure, interference from the host cell proteins, or tedious denaturation and renaturation processes are inevitable, SHTXTHHly offers a good chance to achieve rapid bioactivity detection.

Currently, most mAbs approved for therapeutic applications are produced in mammalian cells since their function relies heavily on correct folding and proper glycosylation, which cannot be supplied by the prokaryotic system [30]. However, *E. coli*, on the other hand, remains a choice for mAb fragments such as Fab, single-chain Fv (scFv), and scFv-Fc fusion [31]. It has been reported that Fcγ receptor I (CD64) can bind to a monomeric de-glycosylated IgG1 Fc variant (mFc) with higher affinity than mammalian cell-expressed IgG1 Fc [17]. Given that the IgG1 antibodies can mediate the CD64 chimeric NK cells to kill tumor cells efficiently [32], we produced the mFc using the SHTXTHHly system and explored its therapeutic potential. Owing to the higher affinity, mFc elicited stronger ADCC and cell activation effects than native Fc. We infer that the overall weak specific lysis effects were attributed to Jurkat cells’ intrinsic weak cytotoxicity. The production of a large amount of IL-2 further proved that once opsonized on the target antigen, mFc-based antibodies evoked the CD64 chimeric effector cells’ strong outputs. The infused mFc-based antibodies may recruit CD64 expressing immune cells, like macrophages, to recognize, endocytose, and clean pathogens. The combination of CD64 chimeric immune cells and mFc-based antibodies may also exhibit considerable antitumor efficacy (Fig. 6). These suggestions show the therapeutic potential of the bacteria-produced antibodies, which would greatly expand the prokaryotic expression system’s application. Besides, these outputs also can be detected with the SHTXTHHly fusion proteins, indicating that the efficacies of the mFc-based antibodies can be quickly and easily evaluated with a tiny amount of culture supernatants.

There are several limitations to our research. The underlying mechanisms for the promotion effect of positive charges and N-terminus fused S tag on secretion level should be thoroughly investigated, as this will allow us to improve secretion efficiency further and better understand the HlyA secretion system. Given the great meaning of the high-level secretion of large proteins in *E. coli*, this is an urgent issue. Moreover, the described system contains extra amino acids from the TEV protease cleavage site, which may alter the chemical and biological properties of target proteins. Different cleavage strategies that leave no extra amino acids in the target proteins, like inteins [33, 34], should be tested. The industrial potential of the SHTXTHHly system should be validated at the fermenter scale. Last but most important, though the bind profiles of mFc for CD64 were comparable to those of wild-type Fc, the efficacy of mFc-based antibodies should be further evaluated in vivo. All of these are important issues for future research.

## Conclusions

In this work, based on our findings that the N-terminus fused S tag and positive charges significantly increased the secretion efficiency of the *E. coli* HlyA system, we developed a high-performance SHTXTHHly system. Three poorly-secreted antimicrobial peptides achieved considerable high secretion levels with profound activities using this system. Furthermore, various POI, including RGDS, IL-15, and alcohol dehydrogenase, were successfully secreted, and their bioactivities were detected in the SHTXTHHly fusion form, providing a fast way to determine the bioactivities of the target proteins directly with the supernatants. Finally, monomeric Fc antibodies produced in this system, whether in the fusion form or not, can bind to Fcγ receptor I (CD64) and mediate the outputs of CD64 chimeric immune cells, further expanding the applications of this powerful secretory expression prokaryotic system.

## Methods

### Construction of plasmids and expression strains

The plasmids and expression strains were constructed as described previously [5]. Briefly, polymerase chain reactions (PCR) were performed to assemble the genes of tag peptides, AMPs, RGDS/RGES, IL-15, ADH, and Fc with SHT. All the constructs were inserted into the pET-THHly plasmid by Gibson Assembly. The *E. coli* DH5α was employed for genetic manipulation, and the *E. coli* BL21 (DE3) was used for protein expression. The expression plasmids and pBAD-BD were co-transformed into BL21 (DE3), and the positive clones were selected by LB agar plate containing Ampicillin (100 μg/mL) and Kanamycin (10 μg/mL). The peptide/protein sequences were listed in Additional file 1: Table S1, S2, and all the plasmids were verified by DNA sequencing. The pI values were calculated in SnapGene 3.2.1 software (Dotmatics, UK).

### Secretory expression and purification of the target proteins

Overnight cultures were inoculated (1% v/v) into a shake flask containing fresh LB media with Ampicillin (100 μg/mL) and Kanamycin (10 μg/mL). Arabinose (0.08% w/v) and IPTG (100 μM) were added 2 h and 6 h after the inoculation, respectively, and the cultures were incubated for another 4 h after the addition of IPTG. The culture condition was maintained at 37 °C and 220 rpm or 20 °C and 150 rpm (for SHTIL15THHly and SHTADHTHHly) after the addition of IPTG.

The cultures were centrifuged at 12,000 g for 3 min. The supernatants were filtered through a 0.45 μm filter and then loaded onto a HisCap Smart 6FF column (Smart-Lifesciences, China) pre-equilibrated with buffer A (50 mM NaH_2_PO_4_, 300 mM NaCl, pH 8.0) with the AKTA Start Protein Purification System (GE Healthcare, USA). After washing the column with 5 column volumes (CV) of buffer A, the fusion protein was eluted with buffer B (50 mM NaH_2_PO_4_, 300 mM NaCl and 200 mM imidazole, pH 8.0). The fusion proteins were digested by His tag conjugated TEV enzyme (Beyotime, China) at 4 °C overnight. The digestion solution was diluted with buffer A and purified by the nickel affinity chromatography again to collect the target proteins in the FT. The final product would be ultra-filtrated to concentrate (3 kDa for rP3 and rL3, and 1 kDa for rA12). SDS-PAGE was used to analyze the loading solution, FT, and eluent fractions. The concentration of the proteins was measured with BCA Quantification Kit.

### Antimicrobial/antitumor activity detection test

Overnight cultures of *E. coli* 25922 were diluted with fresh Mueller–Hinton Broth (MHB) to an optical density at 600 nm (OD_600_) at 0.1 and further diluted at 1:100 with fresh MHB. Then 100 μL bacterial suspension was added in triplicate to the 96-well plate. The commercial (APeptides, China) and bio-produced Cecropin A or LL37 were serially diluted with MHB medium and added into the 96-well plate to final concentrations of 2 to 2000 nM. The plate was incubated at 37 °C for 18 h, and the OD_600_ values were measured with Synergy LX multi-mode reader (BioTek, USA). Broth with bacterial inoculum without AMP and broth alone were used as control groups in which the relative biomass was set as 1 and 0, respectively.

Ten thousand U87 MG cells were seeded in 96-well plates for 16 h to reach the log growth phase and then incubated in triplicate with commercial (APeptides, China) or bio-produced Aurein 1.2 (0–64 μM) for each group. After 8 h incubation, 10 μL of Cell Counting Kit-8 (CCK-8) solution (Dojindo, Japan) was added to each well of the plate and further incubated for 4 h. The absorbance was measured at 450 nm. After U87 MG cells were incubated with 10 μM A12 for 4 h, the apoptosis was assessed by Fluorescence-activated Cell Sorting (FACS) (CytoFlex S, Beckman Coulter, USA) using an Annexin V-FITC/PI kit (Vazyme, China). Quantitative analysis of apoptotic cells was carried out using BD Flowjo VX software (Flowjo, BD, USA).

### Bioactivities determination of RGDS, IL-15, and ADH fusion proteins

U87 MG cells were incubated in 200 μL PBS containing 10 μL supernatant of SHTRGDSTHHly or SHTRGESTHHly at 4 °C for 30 min, then stained with mouse anti-S tag antibody and secondary goat anti-mouse IgG (PE), finally measured by FACS. To examine the ability of fusion proteins to inhibit cell attachment, plates were coated with 10 μg/mL fibronectin for 2 h, followed by 10 mg/ml of heat-denatured bovine serum albumin (BSA) for 1 h to cover any nonspecific binding sites. Twenty thousand U87 MG cells were incubated in 100 μL DMEM and 1 μL supernatant of SHTRGDSTHHly or SHTRGESTHHly at 4 °C for 30 min, then transferred into the 96-well plate. At different time points, the unattached cells were removed by two washes with PBS, and the attached cells were subjected to a CCK-8 reagent for quantification. In other experiments, cells were stained with DiO dye (green) and observed with an Olympus CKX53 fluorescence microscope (Olympus, Japan).

Mo7e cells were plated in a 96-well plate with a cell number of twenty thousand per well. After 4 h incubation, serially diluted IL-15 and SHTIL15THHly were added. After 4 days of incubation, the proliferation of cells was measured using the CCK-8 reagent.

The method of ADH activity assay was described previously [35]. Briefly, the reaction system was designed to include 0.5 μmol Ethyl Acetoacetate, 0.05 μmol nicotinamide adenine dinucleotide (NADH), 10 μmol PBS (pH 7.4), and 1 pmol enzyme in a total volume of 100 μL. The activity was measured at 340 nm using a UV spectrophotometer to monitor the decrease in the absorbance of NADH quantitatively. One unit (U) of enzyme activity is defined as the amount of enzyme that catalyzes the oxidation reaction of 1 μmol NADH within 1 min.

### Bioactivities determination of the Fc fusion proteins

The site mutations were introduced by overlap PCR, and the expression and purification procedure of the Fc fusion proteins was the same as above. To measure the binding affinities, we incubated 2E5 JK-64CAR cells with 7 nM target proteins in 200 μL PBS at 4 °C for 30 min. After washing twice with PBS, cells were stained with goat anti-human IgG (APC) (Yeasen, China) at 4 °C for 20 min and then analyzed by FACS.

U87 MG cells were plated in a 96-well plate with a cell number of 5E3 per well, and 7 nM mFc-based antibodies were added after the cells were completely attached. For the fusion proteins, the experiments were conducted with the supernatants directly. After 1 h incubation, 25E3 JK-64CAR (E: T ratio = 5) cells were added to a final volume of 100 μL 1640, and the plate was incubated for another 4 h. The specific lysis was measured with a Lactate Dehydrogenase Cytotoxicity Assay Kit (Beyotime, China). The percentage of specific lysis was calculated as: (experimental release—spontaneous release) / (maximal release -spontaneous release) * 100 (%).

Cell activation and IL-2 release were measured under the same condition, except that the JK-64CAR cells were stained with 5 μM CFSE, fetal bovine serum (FBS) was added to a final concentration of 10%, and the incubation time was elongated to 24 h. The levels of IL-2 in culture supernatants were measured using an enzyme-linked immunosorbent assay (ELISA) kit (Multi Sciences, China), and the CFSE-stained JK-64CAR cells were analyzed by FACS.

### Statistical analysis

All results were presented as the mean ± standard deviation. Statistical analysis was performed by 1-tailed Student’s t-test to identify significant differences unless otherwise indicated. Differences were considered significant at a P value of less than 0.05.

## Supplementary Information

**Additional file 1**: **Figure S1.** Intracellular expression of the tag fusion proteins. SDS-PAGE analysis of culture samples. The tag fusion proteins are indicated above the image. The arrows indicate the tag fusion proteins. **Figure S2.** Thermal stability of the bio-produced AMPs. *E. coli* 25922 cells were spread over the LB agar plates containing filter-sterilized or autoclave-sterilized bio-produced PEW300/LL37, and incubated at 37 °C for 18 hrs. Nothing was added in the control group. **Figure S3.** Expression and purification of the RGDS (**A**), IL-15 (**B**), and ADH (**C**) fusion proteins. SDS-PAGE analysis of culture samples. W: Whole-cell lysate; S: Supernatant. IB: Inclusion body; So: Soluble proteins; S: Supernatant; FT: Flow-through; E: Eluent. The fusion proteins are indicated above the image. The arrows indicate the target fusion proteins. **Figure S4.** Dimerization determination of the HFc and H’mFc proteins. The sample of the HFc and H’mFc proteins were heated (95 °C, 10 min) or non-heated, then analyzed by SDS-PAGE under reducing or non-reducing conditions. NR: Non-reducing; R: Reducing; H: Heating; NH: Non-heating. **Figure S5.** ADCC effects mediated by bacteria-produced antibodies. The specific lysis for bacteria-produced antibodies opsonized U87 MG cells were detected by LDH Cytotoxicity Assay Kit after 4 hrs of co-incubation (E:T ratio=5). The 1-tailed Student's t-test was used to determine statistical differences between two groups. **Table S1.** Peptides/proteins used in this study. **Table S2.** Site mutated tags used in this study.

## Data Availability

All data generated or analyzed during this study are included in this published article.

## Abbreviations

HlyA: Hemolysin A
TEV: Tobacco etch virus
AMPs: Anti-microbial peptides
mAb: Monoclonal antibody
ADCC: Antibody-dependent cell cytotoxicity
ADH: Alcohol dehydrogenase
ABC: ATP-binding cassette
IPTG: Isopropyl-β-D-thiogalactoside
SDS-PAGE: Sodium-dodecyl sulfate–polyacrylamide gel electrophoresis
FT: Flow-through
MIC: Minimum inhibitory concentration
PBS: Phosphate-buffered saline
kDa: Kilo-dalton
IgG: Immunoglobulin G
CFSE: Carboxyfluorescein succinimidyl ester
ABC: ATP-binding cassette
PCR: Polymerase chain reaction
CV: Column volumes
MHB: Mueller–hinton broth
OD_600_: Optical density at a wavelength of 600 nm
CCK-8: Cell counting kit-8
FACS: Fluorescence-activated cell sorting
BSA: Bovine serum albumin
NADH: Nicotinamide adenine dinucleotide
FBS: Fetal bovine serum
ELISA: Enzyme-linked immunosorbent assay
