# Supplementary material for: SHTXTHHly, an extracellular secretion platform for the preparation of bioactive peptides and proteins in Escherichia coli
Source: Microb Cell Fact. 2022 Jun 27;21:128. doi: 10.1186/s12934-022-01856-8 (PMC9235172; doi:10.1186/s12934-022-01856-8)
Supplement: Supplementary file 1 — Additional file 1: Figure S1. Intracellular expression of the tag fusion proteins. SDS-PAGE analysis of culture samples. The tag fusion proteins are indicated above the image. The arrows indicate the tag fusion proteins. Figure S2. Thermal stability of the bio-produced AMPs. E. coli 25922 cells were spread over the LB agar plates containing filter-sterilized or autoclave-sterilized bio-produced PEW300/LL37, and incubated at 37 °C for 18 hrs. Nothing was added in the control group. Figure S3. Expression and purification of the RGDS (A), IL-15 (B), and ADH (C) fusion proteins. SDS-PAGE analysis of culture samples. W: Whole-cell lysate; S: Supernatant. IB: Inclusion body; So: Soluble proteins; S: Supernatant; FT: Flow-through; E: Eluent. The fusion proteins are indicated above the image. The arrows indicate the target fusion proteins. Figure S4. Dimerization determination of the HFc and H’mFc proteins. The sample of the HFc and H’mFc proteins were heated (95 °C, 10 min) or non-heated, then analyzed by SDS-PAGE under reducing or non-reducing conditions. NR: Non-reducing; R: Reducing; H: Heating; NH: Non-heating. Figure S5. ADCC effects mediated by bacteria-produced antibodies. The specific lysis for bacteria-produced antibodies opsonized U87 MG cells were detected by LDH Cytotoxicity Assay Kit after 4 hrs of co-incubation (E:T ratio=5). The 1-tailed Student's t-test was used to determine statistical differences between two groups. Table S1. Peptides/proteins used in this study. Table S2. Site mutated tags used in this study. [file 12934_2022_1856_MOESM1_ESM.docx]

**Additional information**


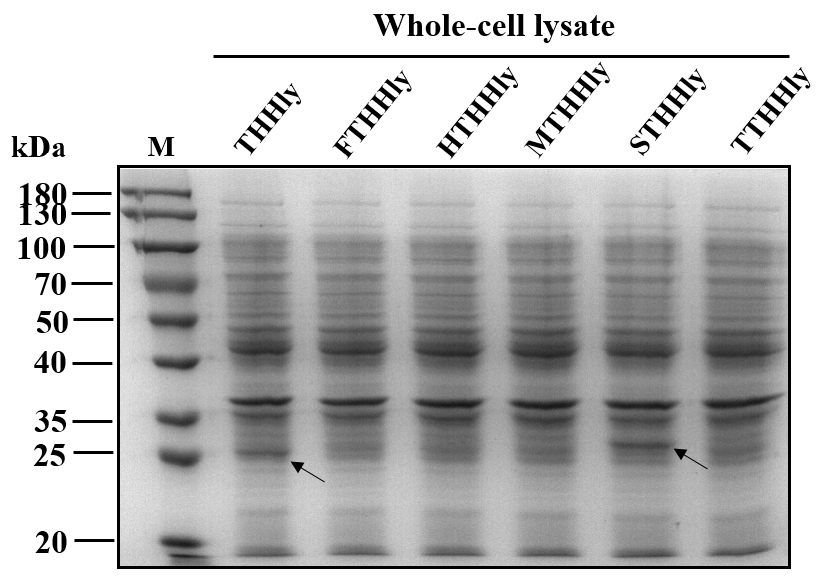


**Figure S1**. Intracellular expression of the tag fusion proteins. SDS-PAGE analysis of culture samples. The tag fusion proteins are indicated above the image. The arrows indicate the tag fusion proteins.


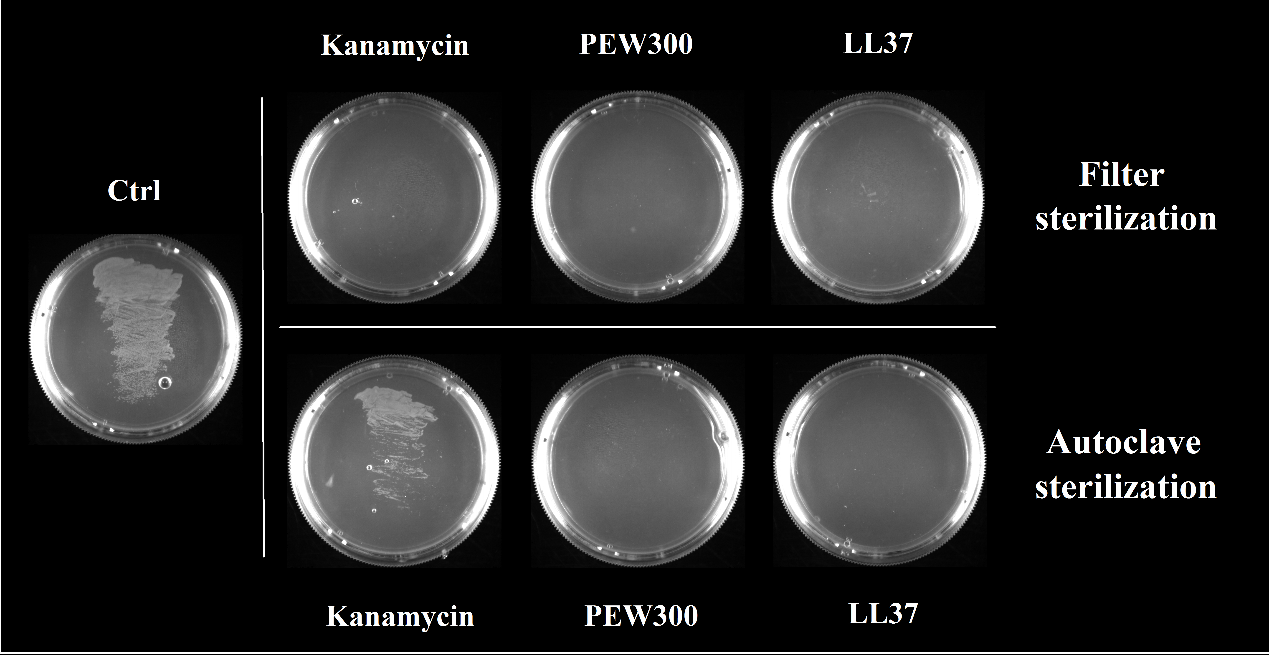


**Figure S2**. Thermal stability of the bio-produced AMPs. *E. coli* 25922 cells were spread over the LB agar plates containing filter-sterilized or autoclave-sterilized bio-produced PEW300/ LL37, and incubated at 37 °C for 18 hrs. Nothing was added in the control group.


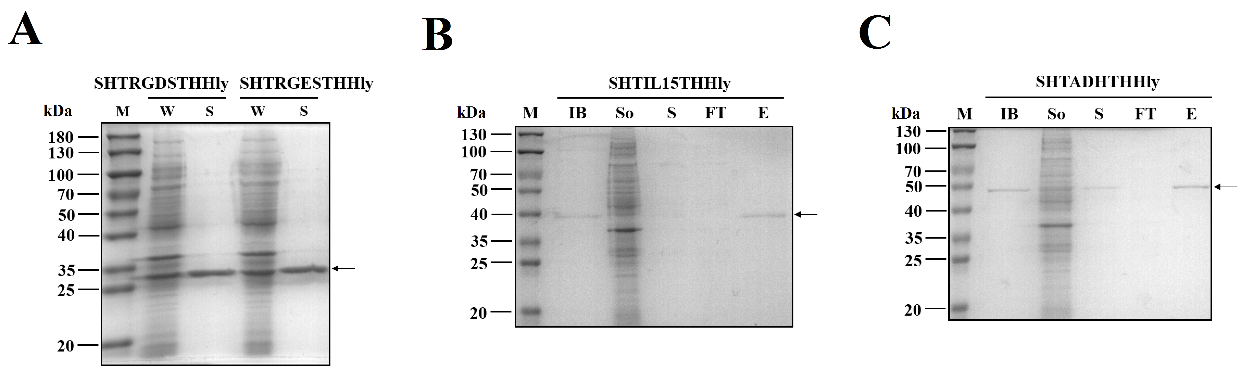


**Figure S3**. Expression and purification of the RGDS (**A**), IL-15 (**B**), and ADH (**C**) fusion proteins. SDS-PAGE analysis of culture samples. W: Whole-cell lysate; S: Supernatant. IB: Inclusion body; So: Soluble proteins; S: Supernatant; FT: Flow-through; E: Eluent. The fusion proteins are indicated above the image. The arrows indicate the target fusion proteins.


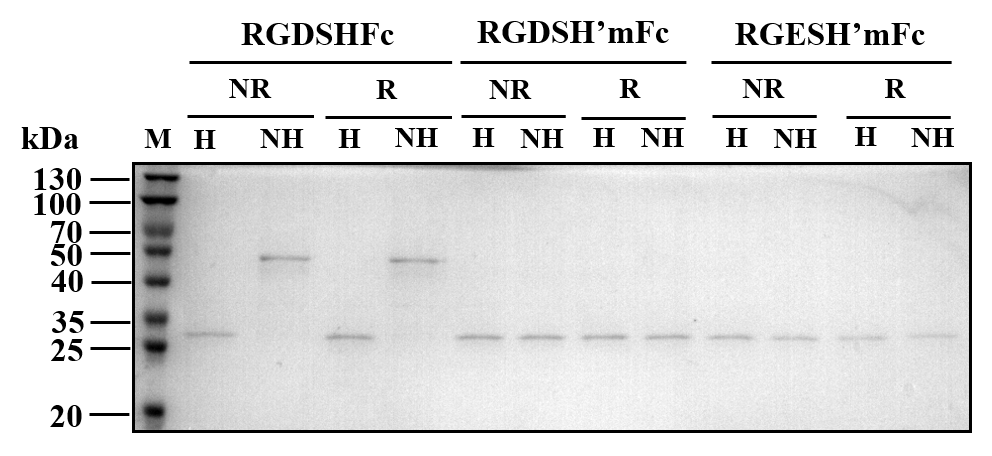


**Figure S4**. Dimerization determination of the HFc and H’mFc proteins. The sample of the HFc and H’mFc proteins were heated (95 °C, 10 min) or non-heated, then analyzed by SDS-PAGE under reducing or non-reducing conditions. NR: Non-reducing; R: Reducing; H: Heating; NH: Non-heating.


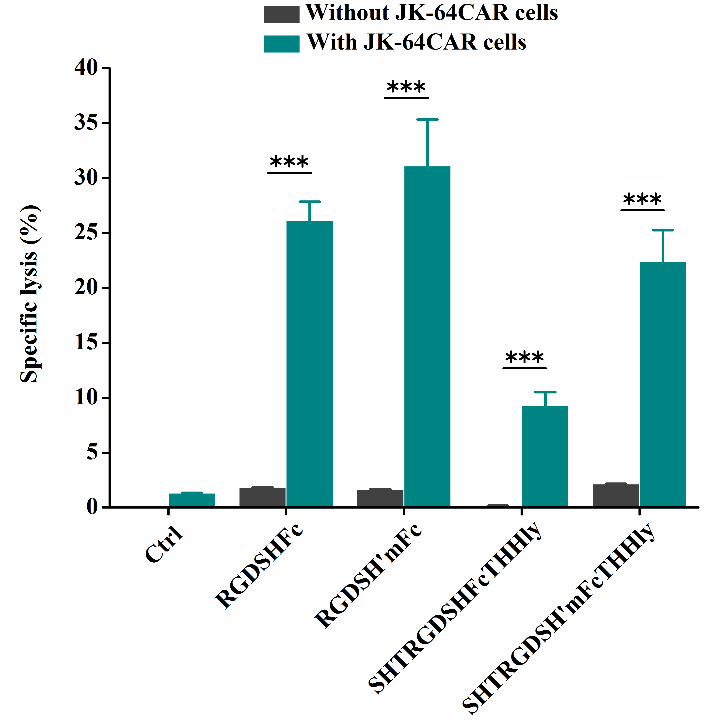


**Figure S5**. ADCC effects mediated by bacteria-produced antibodies. The specific lysis for bacteria-produced antibodies opsonized U87 MG cells were detected by LDH Cytotoxicity Assay Kit after 4 hrs of co-incubation (E: T ratio=5). The 1-tailed Student's t-test was used to determine statistical differences between two groups.

**Table S1**. Peptides/proteins used in this study

| **Peptides/proteins** | **Amino acids sequence** |
| --- | --- |
| Flag tag | DYKDDDDK |
| Myc tag | EQKLISEEDL |
| T7 tag | MASMTGGQQMG |
| His tag | HHHHHH |
| S tag | KETAAAKFERQHMDS |
| Cecropin A | KWKLFKKIEKVGQNIRDGIIKAGPAVAVVGQATQIAK |
| PEW300 | KWKLFKKIHKVGQNIRKGIIKAGPAVAVVGQATQIAK |
| LL37 | LLGDFFRKSKEKIGKEFKRIVQRIKDFLRNLVPRTES |
| Aurein 1.2 | GLFDIIKKIAESF |
| IL-15 | NWVNVISDLKKIEDLIQSMHIDATLYTESDVHPSCKV  TAMKCFLLELQVISLESGDASIHDTVENLIILANDSLS  SNGNVTESGCKECEELEEKNIKEFLQSFVHIVQMFINTS |
| ADH | MIDYQLTGKTAIVTGGVSGIGLAVAQTLAASGARISVWDLKQDAVDATVA  QLRSAGTQAIGIALDVTDDAAVEAAVQRTIKELNGLHVAVNNAGISGP  AASSGDYPIDGWQRVIDVNLTSVFLCQRAQIQAMRAAGTGGSIINMASIL  GQVGYAGSTAYVAAKHGVVGLTQTAAWEHAGDGIRVNAVGPGFISTPLLEKMD  PKVRATLEGRHALKRLGTAEEVAALVAWLASDDASFATGTYYAIDGGYLAQ |
| Fc | APELLGGPSVFLFPPKPKDTLMISRTPEVTCVVVDVSHEDPEVKFNWYVDGVEVHN  AKTKPREEQYNSTYRVVSVLTVLHQDWLNGKEYKCKVSNKALPAPIEKTISK  AKGQPREPQVYTLPPSRDELTKNQVSLTCLVKGFYPSDIAVEWESNGQPENNYKTTPP  VLDSDGSFFLYSKLTVDKSRWQQGNVFSCSVMHEALHNHYTQKSLSLSPGK |
| Monomeric Fc  (The four mutation sites  are highlighted by  bold and underline) | APELLGGPSVFLFPPKPKDTLMISRTPEVTCVVVDVSHEDPEVKFNWYVDGVEVHN  AKTKPREEQYNSTYRVVSVLTVLHQDWLNGKEYKCKVSNKALPAPIEKTISKAKG  QPREPQVYT**S**PPSRDELTKNQVSL**R**C**H**VKGFYPSDIAVEWESNGQPENNYKTT**K**PVL  DSDGSFFLYSKLTVDKSRWQQGNVFSCSVMHEALHNHYTQKSLSLSPGK |

**Table S2**. Site mutated tags used in this study

| Tag | Amino acids sequence | PI value |
| --- | --- | --- |
| F(DD) tag | DYK**D**D**D**DK | 3.47 |
| F(DK) tag | DYK**D**D**K**DK | 4.07 |
| F(KK) tag | DYK**K**D**K**DK | 8.71 |
| M(EE) tag | EQKLIS**EE**DL | 3.68 |
| M(RE) tag | EQKLIS**RE**DL | 4.37 |
| M(RR) tag | EQKLIS**RR**DL | 9.10 |
| T(GG) tag | MASMT**GG**QQMG | 5.50 |
| T(ED) tag | MASMT**ED**QQMG | 3.30 |
| T(RK) tag | MASMT**RK**QQMG | 11.40 |
| S(KK) tag | **K**ETAAA**K**FERQHMDS | 7.16 |
| S(DE) tag | **D**ETAAA**E**FERQHMDS | 3.82 |

(The mutation sites are highlighted by bold and underline)
